# Excitation of delocalized long-lived states of aliphatic protons at low and high magnetic fields

**DOI:** 10.5194/mr-7-81-2026

**Published:** 2026-06-22

**Authors:** Sebastiaan Van Dyck, Coline Wiame, Kirill F. Sheberstov, Geoffrey Bodenhausen

**Affiliations:** 1 Chimie Physique et Chimie du Vivant (CPCV, UMR 8228), Département de Chimie, École Normale Supérieure, PSL University, Sorbonne Université, 75005 Paris, France

## Abstract

Long-lived states (LLSs) can be excited in geminal protons of aliphatic chains by mono- or poly-chromatic spin-lock-induced crossings (SLICs), i.e., by application of one or more selective radio frequency (RF) fields, to create delocalized population imbalances between states belonging to different symmetry under spin permutations. At low fields (in this work at 1.4 T or 60 MHz for proton NMR), these experiments are challenging due to the proximity of the chemical shifts and the need to consider the full untruncated J -coupling Hamiltonian. Five molecules were studied in this work: ethanolamine, lysine, vitamin B1, metronidazole, and phenoxyethylamine (POEA). For POEA and metronidazole, the LLSs are reported for the first time. Measurements were carried out at low and high magnetic fields (1.4 and 11.7 T or 60 and 500 MHz for protons) using 60 MHz Magritek and 500 MHz Bruker NEO spectrometers. The rates $R_{\mathrm{LLS}}=1/T_{\mathrm{LLS}}$ and $R_{1}=1/T_{1}$ were determined using monochromatic SLIC excitation at both fields. We describe strategies for optimizing SLIC conditions in cases where the signals of neighboring CH_2_ groups are relatively close to each other.

## 1 Introduction

**Figure 1:**
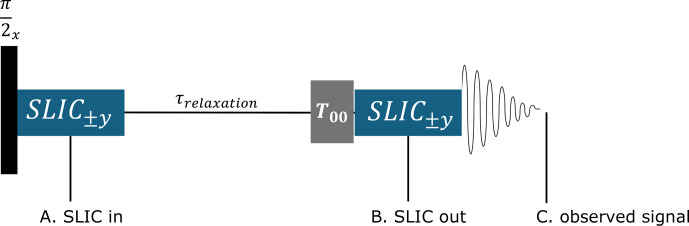
Sequence for the measurement of the relaxation times $T_{\mathrm{LLS}}$ of long-lived states (LLSs) of protons in aliphatic chains comprising $AA^{\prime }XX^{\prime }$ or $AA^{\prime }BB^{\prime }$ systems. The π/2 pulse brings the magnetization into the transverse plane. The first spin-lock-induced-crossing (SLIC) pulse converts this magnetization into an LLS. This pulse is followed by a variable delay and a $T_{00}$ filter that retains only singlet order, while the second SLIC pulse reconverts the LLS into observable magnetization. Cycling of the RF phases along the ±y axes eliminates undesirable signals (Kiryutin et al., 2016). In $AA^{\prime }XX^{\prime }$ or $AA^{\prime }BB^{\prime }$ systems, the SLIC pulses must be applied on resonance with $AA^{\prime }$ , $BB^{\prime }$ , or $XX^{\prime }$ spins.

A long-lived state (LLS) is a nuclear spin state that has a lifetime longer than the longitudinal relaxation time (Carravetta and Levitt, 2004). Usually, an LLS corresponds to an imbalance between states with different spin permutation symmetries (Stevanato et al., 2015; Sheberstov et al., 2019; Sabba et al., 2022). In an isolated two-spin system with two protons H_*A*_ and H_*A* ^′^_, such an imbalance can occur between the average population of three symmetric triplet states ( $|T_{1}\rangle$ , $|T_{0}\rangle$ , $|T_{-1}\rangle$ ) and the population of the singlet state ( $|S_{0}\rangle$ ), which is antisymmetric under spin permutation. The resulting population imbalance is immune to relaxation due to the dipole–dipole coupling between the two protons H_*A*_ and H_*A* ^′^_, thus resulting in a long-lived state. In short *achiral* aliphatic chains – (CH_2_–CH_2_) – with four protons, the geminal proton pairs are chemically equivalent because of the lack of stereogenic centers, but they can be magnetically inequivalent provided each CH_2_ group has a distinct chemical shift and provided the vicinal scalar couplings between neighboring CH_2_ groups differ. This occurs if the populations of the rotamers that result from rotations about the C–C bond are *not* equal so that the differences between the vicinal couplings $\Delta J=J_{AX}$ – $J_{AX^{\prime }}=J_{A^{\prime }X}-J_{A^{\prime }X^{\prime }}$ do not vanish. Magnetic inequivalence allows one to excite an LLS that is *delocalized* across the two $AA^{\prime }$ and $XX^{\prime }$ spin pairs. This can be achieved by mono- or poly-chromatic spin-lock-induced crossings (SLICs) (DeVience et al., 2013; Sonnefeld et al., 2022a, b), i.e., by application of one or two selective radio frequency (RF) fields simultaneously. Although long-lived state excitation can, alternatively, also be achieved via adiabatic-passage spin order conversion (APSOC, Pravdivtsev et al., 2016), this work focuses exclusively on mono-chromatic SLIC excitation. At high fields (e.g., 500 MHz), the RF amplitude for single-quantum (SQ) conditions must be $\nu_{\mathrm{SLIC}}^{\mathrm{SQ}}=2J_{\mathrm{intra}}$ , where $J_{\mathrm{intra}}$ is an averaged value of the intrapair couplings between geminal protons, e.g., $J_{\mathrm{intra}}=\frac{1}{2}\left\{{}^{2}J\left(H_{A},H_{A^{\prime }}\right){+}^{2}J\left(H_{X},H_{X^{\prime }}\right)\right\}$ . A pulse duration $\tau_{\mathrm{SLIC}}^{\mathrm{SQ}}=1/(|\sqrt{2}\Delta J|)$ allows one to achieve SQ level anti-crossing (LAC). After a variable relaxation delay $\tau_{\mathrm{rel}}$ , one applies a $T_{00}$ filter which removes all terms other than the desired population imbalance (Tayler, 2020). A second SLIC pulse then reconverts the LLS into observable magnetization (Fig. 1).

**Figure 2:**
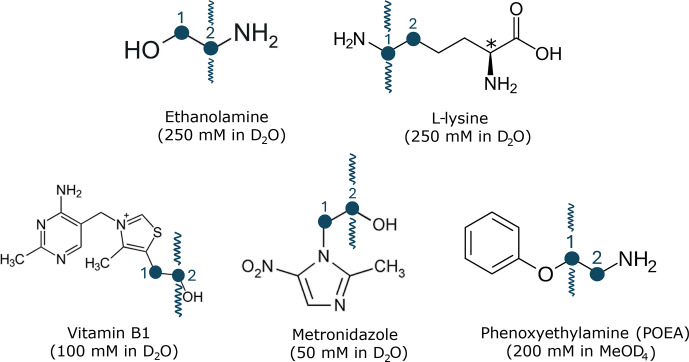
Five molecules where long-lived states have been excited efficiently at both low and high static fields of 1.4 and 11.7 T (60 and 500 MHz for protons). All molecules feature chemically equivalent but *magnetically* *inequivalent* proton pairs of $AA^{\prime }XX^{\prime }$ at high field and $AA^{\prime }BB^{\prime }$ at low field. The wavy arrows indicate the CH_2_ groups that were irradiated in these experiments to excite the LLS by mono-chromatic SLIC (arrows above the molecules) and to reconvert the LLS into magnetization (arrows below the molecules). Note that one can also reconvert LLS on the adjacent CH_2_ group. The relaxation rates $R_{1}=1/T_{1}$ of the CH_2_ groups were determined by the conventional inversion–recovery method. All ligands were dissolved in D_2_O at concentrations in the range between 50 and 250 mM, except for POEA, which was dissolved in MeOD_4_. The samples were not buffered. The pH values are 11.70 for ethanolamine, 6.00 for L-lysine, 2.70 for vitamin B1, 7.15 for metronidazole, and 10.65 for POEA.

Achiral aliphatic chains with suitable four-spin systems are found in ethanolamine, lysine, vitamin B1, metronidazole, and phenoxyethylamine (POEA) (Fig. 2). At high field (e.g., at 11.7 T or 500 MHz for protons), all aliphatic chains in Fig. 2 can be described as $AA^{\prime }XX^{\prime }$ systems in Pople's notation. On the other hand, at low field (e.g., at 1.4 T or 60 MHz), these systems must be described by $AA^{\prime }BB^{\prime }$ to account for the second-order couplings. We show that LLS in these molecules can be excited efficiently at 1.4 T despite the strong coupling regime, provided one re-optimizes the SLIC sequences.

## 2 Methods

### 2.1 Strong coupling at low field

As previously reported (Sonnefeld et al., 2022a, b), the Hamiltonian of a four-spin $AA^{\prime }XX^{\prime }$ system at high magnetic fields (in this work, at 11.7 T) only features strong couplings between the geminal pairs (e.g., H_*A*_ couples strongly to H_*A* ^′^_) but not between the vicinal protons (H_*A*_ couples weakly to H_*X*_). Strong coupling is defined by Δδ<J , whereas weak coupling holds when Δδ≫J . For a four-spin system, Δδ is defined by the difference in chemical shift between the two adjacent CH_2_ spin pairs $AA^{\prime }$ and $XX^{\prime }$ .

**Figure 3:**
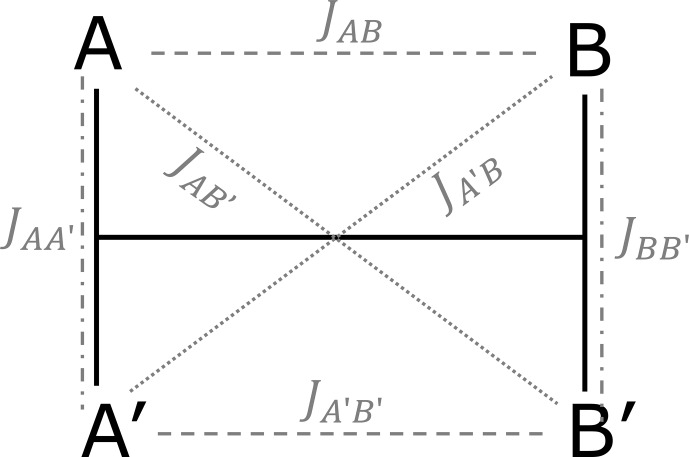
Topological representation of a four-spin $AA^{\prime }BB^{\prime }$ system.

The J couplings are constant. However, Δδ scales with the magnetic field; therefore, as we move to a lower magnetic field, Δδ decreases, which results in a higher ratio of J with respect to Δδ . Therefore, the couplings between geminal proton spin pairs become stronger. That is why the Hamiltonian at low magnetic field (i.e., 1.4 T) may be represented by the topological diagram shown in Fig. 3, where the geminal couplings $J_{AA^{\prime }}$ and $J_{BB^{\prime }}$ are approximately equal, while the vicinal couplings are pairwise degenerate $J_{AB}=J_{A^{\prime }B^{\prime }}$ and $J_{A^{\prime }B}=J_{AB^{\prime }}$ .

The Hamiltonian in units of Hz is

1 $$\begin{array}{rl} H & =\nu_{AA^{\prime }}\left({\hat{I}}_{Az}+{\hat{I}}_{Az{}^{\prime }}\right)+\nu_{BB^{\prime }}\left({\hat{I}}_{Bz}+{\hat{I}}_{Bz{}^{\prime }}\right)\\ & +J_{AA{}^{\prime }}{\hat{I}}_{A}\cdot {\hat{I}}_{A^{\prime }}+J_{BB^{\prime }}{\hat{I}}_{B}\cdot {\hat{I}}_{B^{\prime }}+J_{AB}{\hat{I}}_{A}\cdot {\hat{I}}_{B}\\ & +J_{AB^{\prime }}{\hat{I}}_{A}\cdot {\hat{I}}_{B^{\prime }}+J_{A^{\prime }B}{\hat{I}}_{A^{\prime }}\cdot {\hat{I}}_{B}+J_{A^{\prime }B^{\prime }}{\hat{I}}_{A^{\prime }}\cdot {\hat{I}}_{B^{\prime }} \end{array}$$

where ${\hat{I}}_{i}$ corresponds to the vector representation of the spin operator of spin i , and the operator ${\hat{I}}_{iz}$ represents the z component of the operator ${\hat{I}}_{i}$ . When switching from strong coupling at low field to weak coupling at high field, the non-secular terms of the vicinal J couplings (but not those due to the geminal couplings) can be dropped.

2 $$\begin{array}{rl} H_{\mathrm{vic}}^{\mathrm{LF}} & =J_{AB}{\hat{I}}_{A}\cdot {\hat{I}}_{B}+J_{AB^{\prime }}{\hat{I}}_{A}\cdot {\hat{I}}_{B^{\prime }}+J_{A^{\prime }B}{\hat{I}}_{A^{\prime }}\cdot {\hat{I}}_{B}\\ & +J_{A^{\prime }B^{\prime }}{\hat{I}}_{A^{\prime }}\cdot {\hat{I}}_{B^{\prime }}\\ H_{\mathrm{vic}}^{\mathrm{HF}} & =J_{AB}{\hat{I}}_{Az}{\hat{I}}_{Bz}+J_{AB^{\prime }}{\hat{I}}_{Az}{\hat{I}}_{Bz^{\prime }}\\ & +J_{A^{\prime }B}{\hat{I}}_{Az^{\prime }}{\hat{I}}_{Bz}+J_{A^{\prime }B^{\prime }}{\hat{I}}_{Az^{\prime }}{\hat{I}}_{Bz^{\prime }} \end{array}$$

### 2.2 Effects of second-order vicinal couplings

In a low static field, a weak RF field applied to H_*A*_ and H_*A* ^′^_ also affects the protons H_*B*_ and H_*B* ^′^_. At high field, these effects are negligible so that monochromatic SLIC is truly selective. At low magnetic fields, we have investigated the effects of second-order couplings for mono-chromatic SLIC excitation using simulations with Spin Dynamica (Bengs and Levitt, 2018), written using the Wolfram Mathematica software package.

In a four-spin system $AA^{\prime }BB^{\prime }$ , the LLS part of the density operator ${\hat{\sigma }}_{\mathrm{LLS}}$ , i.e., the population imbalances, always comprises three terms, regardless of how one excites the LLS (Sonnefeld et al., 2022b):

3 $$\begin{array}{rl} {\hat{\sigma }}_{\mathrm{LLS}}= & \lambda_{\mathrm{LLS}}(-\frac{1}{3}{\hat{I}}_{A}\cdot {\hat{I}}_{A^{\prime }}-\frac{1}{3}{\hat{I}}_{B}\cdot {\hat{I}}_{B^{\prime }}+\frac{8}{9}\;\left({\hat{I}}_{A}\cdot {\hat{I}}_{A^{\prime }}\right)\\ & \cdot \left({\hat{I}}_{B}\cdot {\hat{I}}_{B^{\prime }}\right)). \end{array}$$

The LLS yields have been simulated for mono-chromatic SLIC irradiation applied to $AA^{\prime }$ . We chose typical values for a four-spin system: $J_{AA^{\prime }}=J_{BB^{\prime }}=-14$ Hz, $J_{AB}=J_{A^{\prime }B^{\prime }}=5$ Hz and $J_{A^{\prime }B}=J_{AB^{\prime }}=9$ Hz; hence, ΔJ=-4 Hz. At high fields, where the secular approximation can be invoked, the optimum RF amplitude for the single-quantum (SQ-LAC) condition is $v_{\mathrm{SLIC}}=|2J_{\mathrm{intra}}|=28$ Hz, and the optimum SLIC duration is $\tau_{\mathrm{SLIC}}=1/(\Delta J\surd 2)=177$ ms (Sonnefeld et al., 2022a).

**Figure 4:**
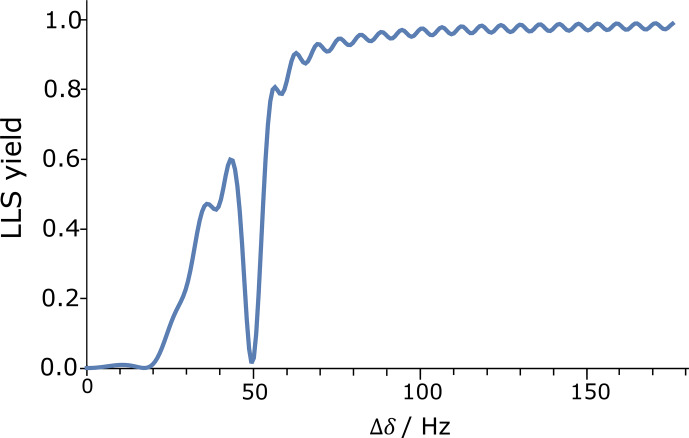
Simulated yields of the excitation of a long-lived state (LLS) as defined in Eq. (3) as a function of the chemical shift difference ( Δδ ) between the $AA^{\prime }$ and $BB^{\prime }$ spin pairs in a four-spin system. Parameters of the SLIC pulse were $v_{\mathrm{SLIC}}=28$ Hz and $\tau_{\mathrm{SLIC}}=177$ ms, corresponding to the high-field SLIC conditions. The LLS yield is normalized to 1 with respect to the high-field regime, which is achieved at the plateau on the right-hand side of the figure.

**Figure 5:**
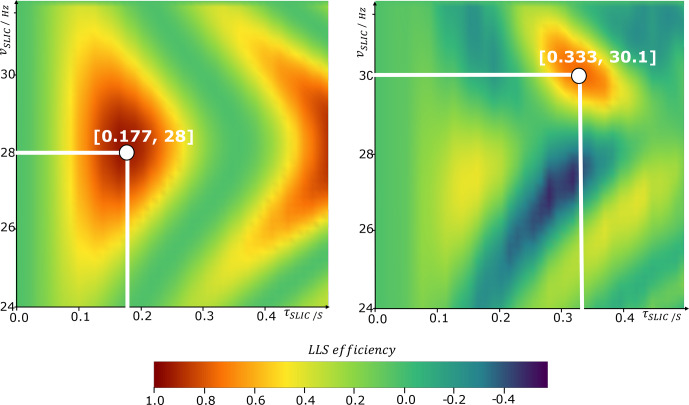
Left panel shows that, for a large difference Δδ=250 Hz, the single-quantum SLIC condition (RF amplitude $v_{\mathrm{SLIC}}$ in the vertical dimension and the duration $\tau_{\mathrm{SLIC}}$ in the horizontal dimension) for a four-spin system ( $v_{\mathrm{SLIC}}=28$ Hz, $\tau_{\mathrm{SLIC}}=177$ ms) match the theoretical conditions at high field ( $v_{\mathrm{SLIC}}=|2J_{\mathrm{intra}}|$ , $\tau_{\mathrm{SLIC}}=1/(\Delta J\surd 2)$ ). However, when the difference is small ( Δδ=52 Hz), the optimum SLIC conditions are $v_{\mathrm{SLIC}}=30.1$ Hz, while $\tau_{\mathrm{SLIC}}=333$ ms. The change in $v_{\mathrm{SLIC}}$ is subtle ( +7 %), but the SLIC duration changes drastically ( +83 %). Since aliphatic – CH_2_ – groups in many of the selected molecules (Fig. 2) have Δδ<60 Hz at 1.4 T, their SLIC duration $\tau_{\mathrm{SLIC}}$ and SLIC amplitude $v_{\mathrm{SLIC}}$ must be re-optimized. The LLS efficiency is normalized to 1 with respect to the high-field regime, which is achieved at the maximum on the left panel.

**Table 1:** Experimentally optimized SLIC conditions for four-spin systems at low (1.4 T) and high (11.7 T) fields. The SLIC amplitude $v_{\mathrm{SLIC}}$ changes for vitamin B1 and metronidazole but remains unchanged for the other ligands. The duration $\tau_{\mathrm{SLIC}}$ increases at a low magnetic field for ethanolamine, vitamin B1, and metronidazole. Note that the reported SLIC conditions for ethanolamine and vitamin B1 deviate from previously reported values (Sonnefeld et al., 2022b) as the molecules in this work were not prepared in buffer. Shifts in pH values can affect SLIC conditions, particularly for ethanolamine. The reported pH values for molecules prepared in D_2_O are as follows: 11.70 for ethanolamine, 6.00 for lysine, 2.70 for vitamin B1, and 7.15 for metronidazole.

Molecule	Δδ (Hz)	$v_{\mathrm{SLIC}}$ (Hz)	$v_{\mathrm{SLIC}}$ (Hz)	$\tau_{\mathrm{SLIC}}$ (ms)	$\tau_{\mathrm{SLIC}}$ (ms)
	at 1.4 T	at 11.7 T	at 1.4 T	at 11.7 T	at 1.4 T
	(60 MHz)	(500 MHz)	(60 MHz)	(500 MHz)	(60 MHz)
Ethanolamine	54	24	25	350	380
Lysine	80	27	27	205	205
Vitamin B1	42	26	27	240	320
Metronidazole	36	26	27	250	310
POEA	61	23	23	240	240

Figure 4 also shows how the LLS yield depends on the chemical shift difference between spins pairs $AA^{\prime }$ and $BB^{\prime }$ . The simulations were done for high-field SLIC conditions ( $v_{\mathrm{SLIC}}=28$ Hz, $\tau_{\mathrm{SLIC}}=177$ ms). At Δδ>60 Hz the LLS yield reaches a plateau that was normalized to 1. The sudden drop in LLS yield – here, at 50 Hz – depends on $J_{\mathrm{intra}}$ ; for higher values of $J_{\mathrm{intra}}$ , the dip shifts to higher frequencies. This means there is a “blind spot” where excitation of LLS cannot be achieved, at least not starting with high-field SLIC parameters. The blind spot can be understood from the dynamics of the off-resonant $BB^{\prime }$ spins in the rotating frame. Although these spins are not meant to be directly addressed by the SLIC irradiation applied to the $AA^{\prime }$ pair, they experience an effective field of magnitude $\nu_{1}^{\mathrm{eff}}=\sqrt{\Delta \delta^{2}+\nu_{\mathrm{SLIC}}^{2}}$ , where Δδ is the frequency offset between the two spin pairs. The dip in the LLS efficiency occurs when this effective nutation frequency matches $2\nu_{\mathrm{SLIC}}$ , which gives the condition $\Delta \delta =\sqrt{3}\nu_{\mathrm{SLIC}}$ .

In Fig. 4, under high-field SLIC conditions ( $v_{\mathrm{SLIC}}=28$ Hz, $\tau_{\mathrm{SLIC}}=177$ ms), the LLS yield is 1.0 for 250 Hz and drops down to 0.35 for Δδ=52 Hz. Figure 4 shows how the LLS yield depends on the chemical shift difference.

The simulations of Fig. 5 show how to optimize the RF amplitude $\nu_{\mathrm{SLIC}}$ and the SLIC duration $\tau_{\mathrm{SLIC}}$ , for the strong coupling regime and single-quantum conditions, to achieve the best LLS yields at different values for Δδ=250 Hz (high-field regime) and Δδ=52 Hz (low-field regime). The figure shows the LLS conversion efficiency normalized to 1 with respect to the high-field regime, which is achieved at the plateau on the right-hand side of the figure. The maximum conversion efficiency in aliphatic spin networks for 4 spin systems for monochromatic SLIC applied to $AA^{\prime }$ spins is achieved when a full population of the $T_{+1}^{AA^{\prime }}T_{0}^{BB^{\prime }}$ or $T_{-1}^{AA^{\prime }}T_{0}^{BB^{\prime }}$ state is transferred to the $S_{0}^{AA^{\prime }}S_{0}^{BB^{\prime }}$ state. This corresponds to ca. 5/72≈7 % population imbalance between the 9 triplet-triplet states and the unique singlet-singlet state.

According to Fig. 5, the LLS yield after re-optimization of $v_{\mathrm{SLIC}}$ and $\tau_{\mathrm{SLIC}}$ is 0.8 for Δδ=52 Hz. We can compare it with the LLS yield in Fig. 4 ( ∼0.35 ) to obtain the enhancement factor. The ratio of the optimized LLS yield to the non-optimized LLS yield is 0.8/0.35≈2.3 .

Subsequently, we re-optimized $\tau_{\mathrm{SLIC}}$ and $v_{\mathrm{SLIC}}$ for each molecule experimentally. The SLIC conditions at 11.7 and 1.4 T are displayed in Table 1, whereas Table 2 shows the improvement in the experimentally achieved LLS yield upon re-optimization of $\tau_{\mathrm{SLIC}}$ and $v_{\mathrm{SLIC}}$ at 1.4 T.

**Table 2:** LLS yield at 1.4 T before re-optimization of $v_{\mathrm{SLIC}}$ and $\tau_{\mathrm{SLIC}}$ (using the conditions listed in columns 3 and 5 in Table 1) and after re-optimization of $v_{\mathrm{SLIC}}$ and $\tau_{\mathrm{SLIC}}$ (using the conditions listed in column 4 and 6 in Table 1). The LLS yield with respect to the thermal signal (when the number of transients and the receiver gain remain the same) is lower than at conventional high field, where the yield is approximately ∼10 % (Sonnefeld et al., 2022b). However, the third column shows that an enhancement, up to a factor of 3.6, has been achieved. This illustrates the need for re-optimization of $v_{\mathrm{SLIC}}$ and $\tau_{\mathrm{SLIC}}$ when Δδ<60 Hz at low magnetic fields. For lysine and POEA, for which the difference in chemical shifts Δδ>60 Hz, the SLIC conditions were identical at 11.7 and 1.4 T, and so no increase in yield was observed.

Molecule	LLS yield	LLS yield	Enhancement factor
	(with respect to thermal)	(with respect to thermal)	(optimized/
	(non-optimized SLIC)/%	(optimized SLIC)/%	non-optimized)
Ethanolamine	5.68	6.25	1.1
Lysine	2.55	2.55	1.0
Vitamin B1	2.12	3.50	1.7
Metronidazole	1.11	4.06	3.6
POEA	6.25	6.25	1.0

**Figure 6:**
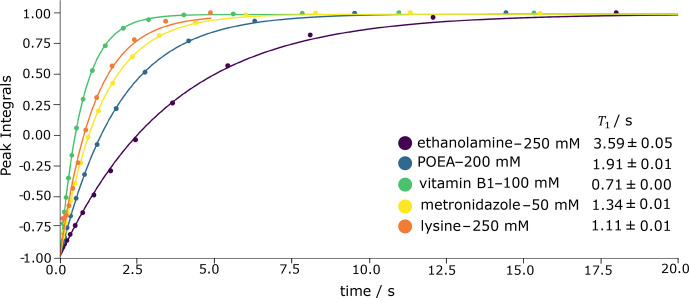
Longitudinal $T_{1}$ relaxation at low field (1.4 T) of the CH_2_ protons highlighted by dots in the five molecules shown in Fig. 2, measured by inversion recovery.

## 3 Comparing $T_{\mathrm{LLS}}$ and $T_{1}$ relaxation time constants at different magnetic fields

The $T_{1}$ and $T_{\mathrm{LLS}}$ of all five molecules shown in Fig. 2 were measured at low and high static fields, in the same sample tubes, at the same concentrations, in the same solvents, and at the same temperatures. The concentrations were chosen to be high to warrant sufficient sensitivity at low field, bearing in mind that the efficiency of two-way (“in-and-out”) SLIC is on the order of only 10 %. In the future we aim to enhance the sensitivity by combining SLIC at both low and high fields with dynamic nuclear polarization (Vasos et al., 2009; Tayler et al., 2012; Bornet et al., 2014; Kiryutin et al., 2019a, b; Razanahoera et al., 2024).

The ratios of the relaxation rates of long-lived states ( $R_{\mathrm{LLS}}=1/T_{\mathrm{LLS}}$ ) and of longitudinal magnetization ( $R_{1}=1/T_{1}$ ), are different at low and high fields (60 and 500 MHz for protons). The ratio $T_{\mathrm{LLS}}/T_{1}$ provides a measure of the usefulness of LLS for various applications such as the measurement of slow motions (Sarkar et al., 2007) or small translational diffusion coefficients (Cavadini et al., 2005).

**Table 3:** $T_{1}$ and $T_{\mathrm{LLS}}$ values at high field (11.7 T).

Molecule	(mM)	CH_2_ group	$T_{1}^{\mathrm{HF}}$ (s)	$T_{\mathrm{LLS}}^{\mathrm{HF}}$ (s)
		(see Fig. 2)	(500 MHz)	(500 MHz)
Ethanolamine	250	2	3.46±0.01	10.33±1.88
Lysine	250	1	1.40±0.01	4.33±0.08
Vitamin B1	100	2	0.73±0.01	5.58±0.27
Metronidazole	50	2	1.27±0.02	3.81±0.14
POEA	200	1	2.21±0.02	9.21±0.49

**Figure 7:**
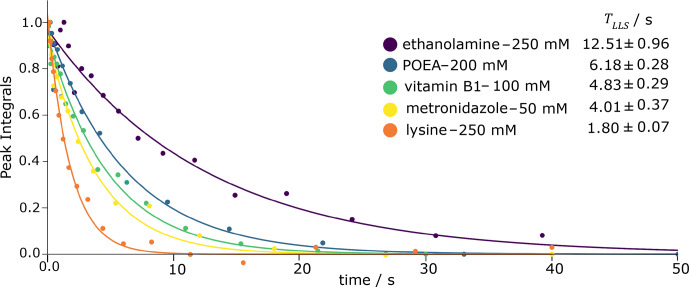
LLS decays at low field (1.4 T) of the aliphatic CH_2_ protons highlighted by curly arrows in the five molecules drawn Fig. 2.

**Table 4:** $T_{1}$ and $T_{\mathrm{LLS}}$ values at low field (1.4 T or 60 MHz for protons).

Molecule	(mM)	CH_2_ group	$T_{1}^{\mathrm{LF}}$ LF (s)	$T_{\mathrm{LLS}}^{\mathrm{LF}}$ (s)
		(see Fig. 2)	(60 MHz)	(60 MHz)
Ethanolamine	250	2	3.59±0.05	12.51±0.96
Lysine	250	1	1.11±0.01	1.80±0.07
Vitamin B1	100	2	0.71±0.00	4.83±0.29
Metronidazole	50	2	1.34±0.01	4.01±0.37
POEA	200	1	1.91±0.01	6.18±0.28

**Table 5:** Ratios $T_{\mathrm{LLS}}/T_{1}$ at high field (11.7 T) and at low field (1.4 T).

Molecule in D_2_O	(mM)	Enhancement	Enhancement	Ratio of
		( $T_{\mathrm{LLS}}/T_{1}{)}^{\mathrm{HF}}$	( $T_{\mathrm{LLS}}/T_{1}{)}^{\mathrm{LF}}$	enhancements
		(500 MHz)	(60 MHz)	HF / LF
Ethanolamine	250	3.0	3.5	0.86
Lysine	250	3.1	1.6	1.94
Vitamin B1	100	7.6	6.8	1.12
Metronidazole	50	3.0	3.0	1.00
POEA	200	4.2	3.2	1.31

## 4 Results and discussion

Inversion–recovery experiments at both low and high fields provided $T_{1}$ values for all samples. The signal integrals of a chosen multiplet (see wavy arrows in Fig. 2) were plotted as a function of the relaxation delay $\tau_{\mathrm{rel}}$ . Figures 6 and 7 show the results obtained at low field. The same $T_{1}$ experiments were repeated at high magnetic field (11.7 T). The results are summarized in Table 3.

To determine the lifetimes $T_{\mathrm{LLS}}$ at low field (1.4 T) by means of SLIC experiments, the delay $\tau_{\mathrm{rel}}$ in Fig. 1 was incremented for each of the five molecules shown in Fig. 2.

Again, these experiments were also carried out with the same samples at high field. The high- and low-field results are shown in Tables 3 and 4. The effect of the magnetic field on the ratio $T_{\mathrm{LLS}}/T_{1}$ is shown in Table 5.

Comparison between the relaxation times $T_{\mathrm{LLS}}$ and $T_{1}$ at high field gives a range $3.0<T_{\mathrm{LLS}}/T_{1}<4.2$ for all molecules except vitamin B1, which has an exceptional gain $T_{\mathrm{LLS}}/T_{1}=7.6$ . At low field, by contrast, the ratios lie in the range of $3.0<T_{\mathrm{LLS}}/T_{1}<6.8$ for all molecules except lysine, which has a rather modest gain $T_{\mathrm{LLS}}/T_{1}=1.6$ . In summary, the $T_{\mathrm{LLS}}/T_{1}$ ratios at high field (11.7 T) are either slightly higher or similar compared to those at low field (1.4 T), except for ethanolamine, where the enhancement is 17 % higher at low field.

## 5 Conclusions

The yield of the excitation of LLS by SLIC at low fields depends on the chemical shift difference Δδ between the neighboring spin pairs. When Δδ≤60 Hz, the pulse amplitude, $\nu_{\mathrm{SLIC}}$ , and duration, $\tau_{\mathrm{SLIC}}$ , must be optimized experimentally, starting at the high-field conditions. The $T_{\mathrm{LLS}}/T_{1}$ ratios at low field (1.4 T) are either slightly lower or similar as at high field.

## Data Availability

The data with inversion recovery and LLS experiments, together with SLIC pulse sequences for Spinsolve Expert, are available at 10.5281/zenodo.20597567 (Sheberstov, 2026a).

## Code availability

The Spin Dynamica codes used to calculate Figs. 4 and 5 are available through the Zenodo repository under 10.5281/zenodo.18684154 (Sheberstov, 2026b).
